# Low to Zero Concentrations of Airborne Bacterial Pathogens and Indicator *E. coli* in Proximity to Beef Cattle Feedlots in Imperial Valley, California

**DOI:** 10.3390/microorganisms11020411

**Published:** 2023-02-06

**Authors:** Xiaohong Wei, Amlan Aggrawal, Ronald F. Bond, Edward R. Atwill

**Affiliations:** Western Center for Food Safety, University of California, Davis, CA 95616, USA

**Keywords:** airborne *E. coli*, STEC, *E. coli* O157, disperse, meteorological data, beef cattle feedlot, risk assessment

## Abstract

This study characterized the effect of distance from beef cattle feedlots, environmental factors, and climate on the occurrence of airborne bacterial indicators and pathogens. Three hundred air samples were collected over 6 months from five feedlots, with each air sample comprising 6000 L of air. Air samples were processed onto TSB-enriched air filters, qPCR-screened, and then qPCR-confirmed for suspect positive colonies of *E. coli* O157, non-O157-Shiga-toxin-producing *E. coli* (STEC), *Salmonella*, and *E. coli*. Direct enumeration of *E. coli* was also collected. Although no bacterial pathogens were qPCR-confirmed for the 300 samples, *E. coli* was detected in 16.7% (50/300) of samples, with an overall mean concentration of 0.17 CFU/6000 L air. Logistic regression analyses revealed a higher odds of *E. coli* for samples in close proximity compared to >610 m (2000 ft) distance from feedlots, along with significant associations with meteorological factors, sampling hour of day, and the presence of a dust-generating activity such as plowing a field or nearby vehicular traffic. The lack of bacterial pathogen detection suggests airborne deposition from nearby feedlots may not be a significant mechanism of leafy green bacterial pathogen contamination; the result of our study provides data to inform future revisions of produce-safety guidance.

## 1. Introduction

During the past five years (2016–2021), there has been one or more outbreaks per year in the United States associated with *E. coli* O157:H7 contamination of leafy greens, based on data from the Centers for Disease Control and Prevention (CDC) [1]. For many of these and earlier foodborne outbreaks of *E. coli* O157:H7 associated with leafy greens, the biological or environmental source of the bacterial contamination was not definitively determined. This ambiguity regarding the underlying source(s) of bacterial contamination has led to a wide range of food safety recommendations and guidance documents in an effort to reduce the risk of pathogen contamination either in the produce field, during harvest and transport, or during processing. A good example is the California Leafy Green Products Handler Marketing Agreement (LGMA) that established food safety metrics with guidance on a wide variety of production management practices and environmental conditions that either enhance or reduce the risk of produce contamination [2]. With respect to this present study in particular, the LGMA offers interim guidance on minimum distances between leafy green production fields and either composting facilities that utilize animal products (122 m or 400 ft) or Concentrated Animal Feeding Operations (CAFOs), with recommended distances of 366 m (1200 ft) from beef cattle feedlots with >1000 cattle and 1609 m (1 mile) for feedlots with >80,000 cattle [2]. The LGMA recognizes that, in many cases, the science needed for data-driven guidance is lacking; hence, many of these food safety recommendations are only interim and subject to revision once the appropriate research has been conducted to better inform their precision, such as the specific distances cited above regarding proximity of a CAFO to a leafy green production field [2,3].

Prior research [3,4,5,6] has been conducted on the relationship between distance from a CAFO (mainly beef cattle feedlots) and the number of microorganisms (*E. coli*, *E. coli* O157) in matrices such as air, soil, or leafy green samples. With respect to air samples, prior research has generally sampled 100 to 1000 liters (L) of air and used culture-based approaches to detect or enumerate microorganisms/L. This body of prior work, while informative, shares the following limitations that our study on airborne transmission attempts to address: only a few discrete distances from the CAFO were evaluated, and there was a narrow scope of target microorganisms, relatively small sample sizes and small sample volumes of air, and a very limited number of feedlots, with some studies not taking into consideration the effect of wind velocity and/or wind direction relative to the CAFO (i.e., upwind or downwind of the CAFO). An additional concern with this prior research is the use of soil or leafy green samples to study airborne deposition of bacteria from a suspected source such as a CAFO; presumably, the bacteria found on or in a sample of native soil or leafy greens in a natural field setting (i.e., uncontrolled environment) is not limited to airborne deposition of planktonic airborne bacteria or from bacteria attached to fugitive dust (i.e., suspended colloids or aggregates) but is the sum total of multiple processes, including but not limited to airborne deposition (as in this this study); arthropods such as house flies or avian species landing, foraging, and/or defecating on the soil or plant surface; and uncontrolled physical contact from other vertebrates when investigators are not present, such as rodents at night.

We conducted the following longitudinal study to determine (1) the baseline prevalence of airborne bacterial pathogens and indicators in proximity to commercial beef cattle feedlots in the seasonal produce production region of Imperial Valley, California; (2) evaluate the effect of LGMA-recommended setback distances from commercial feedlots on the prevalence of airborne bacterial pathogens and bacterial indicators; and (3) quantify the association between environmental factors such as wind speed and relative humidity in regard to the prevalence of airborne bacterial pathogens and indicators. Achieving these goals would allow for a better assessment of the current food safety risks of produce production in proximity to commercial CAFOs for this agricultural region and provide needed data to revise, as needed, the LGMA metrics regarding setback distances between CAFOs such as beef cattle feedlots and fields of preharvest produce, such as lettuce and spring mix.

## 2. Materials and Methods

### 2.1. Site Selection

**Figure 1:**
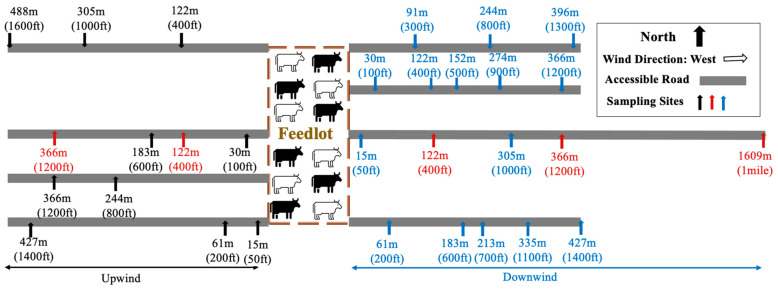
An example distribution of sampling sites with proximity of each feedlot when the wind comes from the west. Red bars, LGMA-fixed sites; blue bars, downwind sites at random distances; green bars, upwind sites at random distances. The thick gray lines represent the publicly accessible roads near feedlot where air sampling was conducted.

We solicited voluntary and confidential participation in this study through in-person meetings in 2019 and 2020 with the feeder cattle industry in Imperial Valley, California. These meetings resulted in the recruitment of five cattle feedlots managed by five different owners from various regions of the county. After discussing the goals of the study with the feedlot owners and confirming their consent to participate, air sampling was conducted on a monthly basis from November 2020 through April 2021. Air sampling typically commenced at ~6:00 a.m. and ended ~3:30 p.m. A total of 300 air samples were collected during the 6 months of field work: 150 air samples collected at the LGMA-guidance distances of ~122 m (~400 ft) from a compost facility and ~366 m (1200 ft) and ~1609 m (1 mile) from a cattle feedlot, as well as an additional 150 samples collected at randomized distances from the edge of the five feedlots ranging from 9 m (30 ft) to 610 m (2000 ft) in any direction (Table 1 and Figure 1). These five feedlots did not endeavor to produce actual compost from their animal manure. Instead, these facilities stacked their dried manure solids from scraped pens and stored the material in windrows in a specific area of the feedlot operation, which, for this project, simulated the risk of fugitive dust from a certified compost operation; hence, we located some of our sample sites ~122 m (400 ft) from these locations. With respect to sample locations at the fixed LGMA-guidance distances and given that the prevailing wind direction is generally from west to east in Imperial Valley for this time of year, we allocated five of these fixed-distance sites to each feedlot: two sites at ~122 and ~366 m west of each feedlot (typically upwind), two sites at ~122 and ~366 m east of each feedlot (typically downwind), and one site ~1609 m (~1 mile) away east of each feedlot (typically downwind). These five sites were repeatedly sampled each month at each of the five feedlots, resulting in a total of 150 LGMA-fixed distance air samples.

In order to complement these LGMA-fixed air sampling sites, we also randomly allocated five sites at various set distances per feedlot per month: two of these five sites were situated upwind, at randomized set distances of either 15 (50), 30 (100), 61 (200), 122 (400), 183 (600), 244 (800), 305 (1000), 366 (1200), 427 (1400), or 488 (1600) m (ft); and three of these five sites were situated downwind, at randomized set distances of either 15 (50), 30 (100), 61 (200), 91 (300), 122 (400), 152 (500), 183 (600), 213 (700), 244 (800), 274 (900), 305 (1000), 335 (1100), 366 (1200), 396 (1300), or 427 (1400) m (ft) (Figure 1). Lack of access at some intended sample sites required an adjustment to as close as possible to the intended distance. The upwind–downwind designation for allocating these random sites was determined by visiting each feedlot one day prior to planned air sampling and recording the predicted next-day prevailing wind direction, using the cell phone application Weather Mate (version 6.4.2), with the final upwind–downwind designation revised as needed during the next day of actual air sampling.

**Figure 2:**
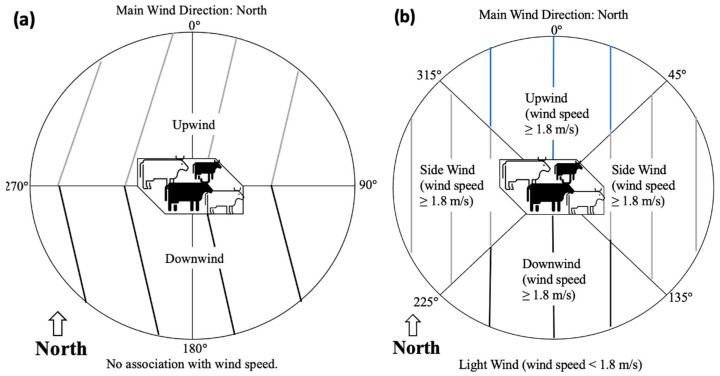
Two different categorizations (**a**,**b**) for indicating position of the air sampler relative to feedlot location and wind direction, with an example when wind comes from the north: (**a**) With prevailing wind direction set at 0°, downwind from a feedlot is a 180° partition of 270° to 90°, and upwind from a feedlot is a 180° partition of 270° to 90°. (**b**) With wind direction set at 0°, downwind from a feedlot is a 90° partition or 135° to 225° when windspeed is ≥1.8 m/s (4 mph); other category includes 90° partitions for upwind (315° to 45°) and both lateral sidewinds (45° to 135° and 225° to 315°) when wind speed is ≥1.8 m/s (4 mph); and light wind is when wind speed is <1.8 m/s (4 mph) regardless of wind direction, position of samplers, and feedlot.

We utilized two different categorizations for indicating the position of the air sampler relative to the feedlot location and wind direction (Figure 2): Figure 2a, with prevailing wind direction set at 0°, downwind from a feedlot is a 180° partition of 270° to 90°, and upwind from a feedlot is a 180° partition of 270° to 90°; Figure 2b, with wind direction set at 0°, downwind from a feedlot is a 90° partition or 135° to 225° when windspeed is ≥1.8 m/s (4 mph), other category includes 90° partitions for upwind (315° to 45°) and both lateral sidewinds (45° to 135° and 225° to 315°) when wind speed is ≥1.8 m/s (4 mph), and light wind is when wind speed is <1.8 m/s (4 mph) regardless of wind direction, position of samplers and feedlot. The cutoff for designating wind as light (<1.8 m/s) was based on a definition used by the U.S. National Weather Service (www.weather.gov/mediaas/pqr/wind/wind.pdf, accessed on 1 July 2022).

### 2.2. Sample Collection

Air samples were collected by using MAS-100 Eco microbial air samplers (Merck KgaA, Darmstadt, Germany), which were factory calibrated prior to the study. For each sampling event, 6000 L of air was collected at a flow rate of 100 L/min (1 h total). Air samplers were attached to portable tripods at a height of 1.2 m (4 ft) above the ground. Before and after sampling at each site, 70% ethanol was sprayed over the samplers, especially the lids, for disinfection.

Two sources of meteorological data were collected, in situ and weather station. For the in situ data, wind speed, wind direction, air temperature, and relative humidity were collected at 30 s intervals during the 1 h of air sampling by using a tripod-mounted Kestrel 5500, which was calibrated daily. Arithmetic means of the meteorological data were then calculated for each site’s sampling event. For weather-station meteorological data, hourly records were obtained from CIMIS (https://cimis.water.ca.gov, accessed on 15 May 2022) based on the closest active weather station to the sampling site location and sampling date and hour.

### 2.3. Microbiological Analysis

**Figure 3:**
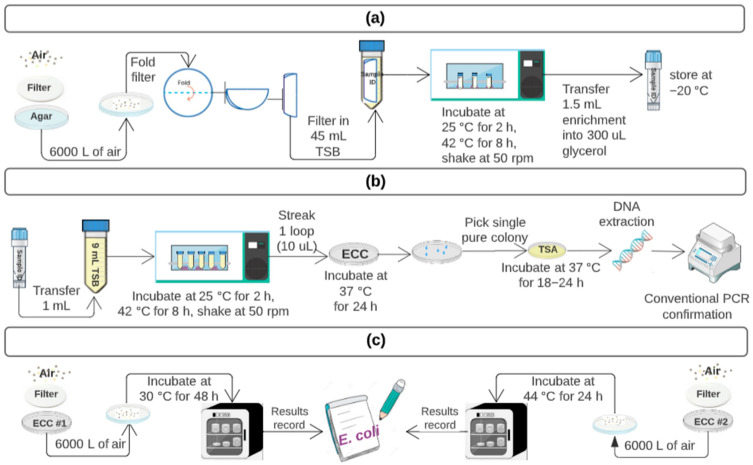
The flowchart for pretreatment of air samples for bacterial detection, and indicator *E. coli* culture methods for direct counts and for enrichments: (**a**) Pretreatment for indicator *E. coli* and pathogens. (**b**) Indicator *E. coli*_Enrichment. (**c**) Indicator *E. coli*_Direct count. Agar = Research Products International Bacteriological Grade Agar, TSB = tryptic soy broth, ECC = CHROMagar ECC, TSA = tryptic soy agar.

For each site, 6000 L of processed air was impinged onto a Whatman Quartz Air Sampling Filter (VWR International, Radnor, CA, USA) [7] that had been placed on top of Research Products International Bacteriological Grade Agar (Neta scientific Inc., Hainesport, NJ, USA) (Figure 3a). Using sterile forceps, filters were folded into 50 mL conical tubes (Fisher Scientific, Pittsburgh, PA, USA) and incubated in 45 mL tryptic soy broth (TSB; Difco, BD, San Jose, CA, USA) on a shaking incubator (50 rpm) at 25 °C for 2 h, followed by 42 °C for 8 h, with 1.5 mL of enrichment then frozen at −20 °C in 300 μL of glycerol. For indicator *E. coli* TSB-enrichment detection, 10 μL loopful of TSB enrichment was streaked onto CHROMagar ECC (ECC; DRG international, Inc., Springfield, NJ, USA), and suspect colonies were confirmed with conventional PCR (Figure 3b) [8,9]. A second 6000 L sample was also collected from the same site at each event and impinged onto ECC for a direct count of indicator *E. coli* (Figure 3c), with all isolates confirmed as *E. coli* by using conventional PCR, as mentioned above for isolates from TSB enrichment.

**Figure 4:**
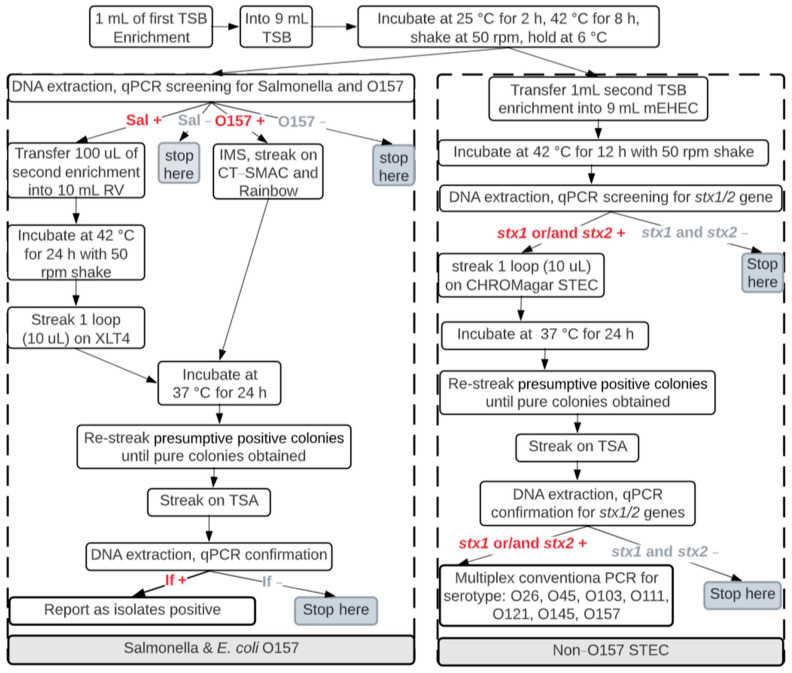
The flowchart for pathogen detection from enrichments (*E. coli* O157:H7, STEC, and *Salmonella*) from air samples. TSB = tryptic soy broth; qPCR = quantitative PCR; mEHEC = Modified Enterohemorrhagic *Escherichia coli* Broth; CT-SMAC = MacConkey Agar with Sorbitol, Potassium tellurite, and Ceffeximine; Rainbow = Rainbow Agar O157; O157 = *E. coli* O157; Sal = Salmonella; STEC = non-O157 Shiga-toxin–producing types of *E. coli*; RV = Rappaport-Vassiliadis, TSA = tryptic soy agar; IMS = Immuno-Magnetic Separation; XLT4 = Xylose lysine Tergatol-4; ECC = CHROMagar ECC; − represents negative, and + represents positive.

For the detection of bacterial pathogens, 1 mL of the initial TSB enrichment was re-enriched. These secondary enrichments of TSB and the enrichment of Modified Enterohemorrhagic *Escherichia coli* Broth (mEHEC; BioControl Systems Inc., Bellevue, WA, USA) were then screened for *E. coli* O157, *Salmonella*, and *stx* 1/2 genes by using quantitative-PCR (qPCR), as described in Figure 4 [10,11,12]. All suspect positives from the qPCR screen were plated onto their respective selective agar (Figure 4): MacConkey Agar with Sorbitol, Potassium tellurite, and Ceffeximine (CT-SMAC; BD BBL, Sparks, MD, USA) and Rainbow Agar O157 (Rainbow; Biolog, Inc., Hayward, CA, USA) for *E. coli* O157; CHROMagar STEC (DRG international, Inc., Springfield, NJ, USA) for STEC; and Xylose lysine Tergatol-4 (XLT4; Neogen Culture Media, Lansing, MI, USA) for *Salmonella*. Any suspect colonies were qPCR-confirmed as previously described [10,13]. Suspect STEC colonies were then confirmed by using multiplex conventional PCR to identify O26, O45, O103, O111, O121, O145, and O157 serogroups of *E. coli* [14].

### 2.4. Statistical Analyses

The linear distances between air sampling sites and the closest edge of a pen of cattle or compost (stacked manure) yard were calculated by SAS University based on GPS (the global positioning system) coordinates. SAS University and R Studio were used to compile the data and perform statistical analysis. Two-sample *t*-test, Chi square, and Fisher’s exact tests were used to analyze the difference between meteorological data obtained from in situ and weather station. Fisher’s exact test was used to compare the prevalence of *E. coli* (enrichment) among different LGMA-guidance distances. Logistic regression was used to identify environmental variables associated with the odds of detecting airborne indicator *E. coli*, using a forward stepping algorithm and either a *p*-value ≤ 0.05 based on a likelihood ratio test or a reduction in the AIC value for inclusion of the variable in the final model.

## 3. Results

### 3.1. Meteorological Conditions during Sampling

We obtained meteorological data from two different sources (in situ Kestrel and nearby commercial weather station) in order to assess the impact of data source on our final statistical models characterizing the association between these meteorological factors and the occurrence of bacterial pathogens and indicators. During days of field sampling in Imperial Valley, the mean and range of in situ and weather-station-obtained data for air temperature was 20.1 °C (6.2–36.4 °C) and 18.4 °C (2.2–36.7 °C), respectively; relative humidity was 37.8% (7.0–100.0%) and 37.7% (9.0–99.0%), respectively; and wind speed was 1.9 m/s (0.1–6.1 m/s) and 2.3 m/s (0.4–8.6 m/s), respectively. There was a significant difference between the data obtained by in situ instruments versus the weather station for air temperature (*p* < 0.001) and wind speed (*p* < 0.001) for the same time periods, but not for relative humidity (*p* = 0.12). For wind direction relative to the location of the air sampler and the feedlot, the agreement between data from the in situ instrument and weather station was 78.0% (234/300) when we used the first categorization for wind direction (180° upwind or downwind of feedlot), which was significantly different (*p* < 0.001). When the second categorization for wind direction was used (90° downwind, light wind, or other (90° sidewind or upwind)), the agreement between in situ and weather station was 57.3% (172/300), which was significantly different (*p* < 0.001). These significant differences between the two sources of meteorological data for the same time periods in Imperial Valley indicate that the final statistical models characterizing the association between these meteorological factors and the occurrence of airborne bacterial pathogens and indicators may be somewhat different.

### 3.2. Detection of Bacterial Pathogens and Indicator E. coli

Indicator *E. coli* was detected and confirmed in 16.7% (50/300) of enriched air samples from all locations, with a mean concentration of 0.17 CFU/6000 L from direct counts. In addition, indicator *E. coli* was detected in 11.3% (17/150) of enriched air samples from sites located at the LGMA-guidance distances of either ~122 m (~400 ft) from stacked animal manure, ~366 m (~1200 ft) distance from cattle feedlots with >1000 head, and ~1609 m (~1 mile) distance from cattle feedlots with >80,000 head, with an overall mean concentration of 0.06 CFU/6000 L from direct counts. Based on enriched samples, the prevalence of indicator *E. coli* ranged from 13% to 32% within 366 m (1200 ft) of a feedlot edge for all 300 samples, and it declined to 8–9% at distances greater than 366 m (1200 ft) (Table 1). Interestingly, with respect to the three LGMA-guidance distances, there was no significant difference (*p* = 0.51) in the prevalence of indicator *E. coli* in enriched air samples taken at ~122 m (~400 ft, 8.3% positive), ~366 m (1200 ft, 15.0% positive), and ~1609 m (~1 mile, 10% positive), with a minor decrease in concentration at increasing distances (0.08, 0.05, and 0.03 CFU/6000 L at ~122 m (~400 ft), ~366 m (~1200 ft), and ~1609 m (~1 mile) distance, respectively, for direct counts). 

None of the 300 air samples (6000 L of air/sample) was positive for bacterial pathogens based on a positive qPCR screen of the enrichment followed by qPCR-confirmation of a suspect colony on selective agar. One air sample screened positive by qPCR for *Salmonella* that was located 396 m (1300 ft) downwind from a feedlot, but no colony was qPCR confirmed for *Salmonella* from selective agar; hence, it was considered to be negative. The other 299 air samples taken in closer or further proximity to the five feedlots screened negative for *Salmonella*, and all 300 samples screened negative for *E. coli* O157.

Sample enrichments from sixteen air samples screened qPCR-positive for the s*tx* 1/2 gene, but none of these samples had suspect STEC colonies confirmed by qPCR for *stx* 1/2 genes (Table 1). These sixteen samples that were positive for *stx* 1/2 genes were located at all distances from the feedlots, with no significant trend in *stx*-gene positivity relative to distance or proximity to the feedlots.

**Table 1:** Prevalence of indicator *E. coli* ^a^, *E. coli* O157 ^b^, non-O157 STEC ^c^, and *Salmonella* ^b^ in 6000-L air samples taken in proximity to five commercial feedlots in Imperial Valley, California (November 2020–April 2021).

Site	Distance from Feedlot Edge Meter (Feet)	Total Air Samples	*E. coli*			qPCR Screen on Enrichments			qPCR Confirmed Isolates		
			Mean (Min–Max)	% Positive	% Positive	% Suspect Positive			% Confirmed Positive		
			Direct Count (CFU/6000 L)		Enrichments (+ or −/6000 L)	*E. coli* O157	*stx* 1/2	*Salm*	*E. coli* O157	STEC *stx 1/2*	*Salm*
All sites (random distance and LGMA-fixed distance)	9–36 (30–120)	22	0.95 (0–19)	13.64%	31.82%	0	4.55%	0	0	0	0
	37–128 (121–420)	79	0.13 (0–5)	5.06%	12.66%	0	3.80%	0	0	0	0
	129–250 (421–820)	47	0.21 (0–8)	6.38%	19.15%	0	12.77%	0	0	0	0
	251–372 (821–1220)	94	0.09 (0–3)	6.38%	20.21%	0	2.13%	0	0	0	0
	373–616 (1221–2020)	26	0.04 0–1)	3.85%	7.69%	0	7.69%	3.85%	0	0	0
	616–1829 (2021–6000)	32	0.03 (0–1)	3.13%	9.38%	0	6.25%	0	0	0	0
	Total	300	0.17 (0–19)	6.00%	16.67%	0	5.33%	0.33%	0	0	0
Fixed sites at LGMA guidance distances	~122 (~400)	60	0.08 (0–3)	5.00%	8.33%	0	5.00%	0	0	0	0
	~366 (~1200)	60	0.05 (0–3)	1.67%	15.00%	0	3.33%	0	0	0	0
	~1609 (~1 mile)	30	0.03 (0–1)	3.33%	10.00%	0	6.67%	0	0	0	0
	Total	150	0.06 (0–3)	3.33%	11.33%	0	4.67%	0	0	0	0
Random distance	9–36 (30–120)	22	0.95 (0–19)	13.64%	31.82%	0	4.55%	0	0	0	0
	37–128 (121–420)	29	0.21 (0–5)	6.90%	24.14%	0	3.45%	0	0	0	0
	129–250 (421–820)	37	0.24 (0–8)	5.41%	18.92%	0	13.51%	0	0	0	0
	(251–372) 821–1220	37	0.14 (0–1)	13.51%	29.73%	0	0	0	0	0	0
	373–616 (1221–2020)	25	0.04 (0–1)	4.00%	4.00%	0	8.00%	4.00%	0	0	0
	Total	150	0.28 (0–19)	8.67%	22.00%	0	6.00%	0.67%	0	0	0

% Positive = positive percentage (prevalence percentage), *Salm = Salmonella*, LGMA = California Leafy Green Products Handler Marketing Agreement. ^a^ Positivity for indicator *E. coli* requires culture positive on selective agar followed by traditional PCR confirmation of the isolate. ^b^ Positivity for *E. coli* O157 and *Salmonella* requires qPCR positive screen from TSB enrichment broth followed by qPCR confirmation of 1 or more isolates from selective agar. ^c^ STEC positivity requires qPCR positive screen for *stx* 1/2 from mEHEC enrichment, followed by qPCR confirmation of one or more isolates from selective agar for *stx* 1/2, followed by a positive multiplex PCR designation for either O26, O45, O103, O111, O121, or O145.

### 3.3. Logistic Regression Models for the Association between Detecting Airborne E. coli and Various Environmental and Seasonal Variables in Proximity to Feedlots

After combining all of the data for indicator *E. coli* from both random and LGMA-fixed sites shown in Table 1, we generated four different regression models (Table 2) in order to compare model inferences from two different sources of wind speed and direction (in situ Kestrel and weather station) and two different methodologies for determining the aspect of the air sampler relative to wind direction and feedlot position (i.e., sampler is upwind or downwind of feedlot, alongside or sidewind, or little to no wind (aspect designation shown in Figure 2 in Section 2)). The four models (A1, A2, A3, and A4) had relatively similar sets of significant variables associated with indicator *E. coli*, with notable exceptions being dust-generating activity (significant only for Models A1 and A2 for in situ meteorological data), wind speed (significant only for Model A3 for weather station meteorological data), and position of air sampler relative to the feedlot and wind direction (lack of significant differences between downwind, light wind, and other for Model A2). The variable “position of air sampler” was retained in regression Model A2 because its inclusion improved the final AIC and Hosmer–Lemeshow GOF. The reason why wind speed was not significant for Models A2 and A4 is because the method of categorization for the location of the air sampler relative to wind direction and feedlot position in these two models included a light-to-no-wind category, effectively taking into account the wind speed within this categorical variable and thereby rendering the continuous variable “wind speed” non-significant (Table 2).

Logistic regression models generate coefficients (β_i_X_i_) for the log odds of detecting a categorical outcome, which, in this study, is the odds of detecting indicator *E. coli* in a 6000 L air sample as a function of various environmental and seasonal variables. The exponentiation of these model coefficients (e^βiXi^ = odds of *E. coli* detection) generates the odds of detecting *E. coli* in an air sample for the specified variable relative to the referent category. For example, for Model A, when dust-generating activity was observed during air sampling (e.g., truck drives nearby on dirt road while air sampling), the odds of detecting *E. coli* was 2.57-times higher (e^0.94^ = 2.57) compared to those of air samples collected when no such activity was observed. Additional interpretations of the regression models in Table 2 are as follows: for each additional hour later in the day when a sample was taken, the odds of detecting *E. coli* was 0.4-times lower compared to samples taken an hour earlier for all four models (i.e., morning samples had a higher prevalence of airborne *E. coli* compared to afternoon samples). For each 10% percent increase (0 to 100 scale) in relative humidity during the time period the air sample was taken, the odds of detecting *E. coli* decreased by 0.74- to 0.67-times for all four models. Relative to distances in excess of 610 m (2000 ft) between the air sampler and the edge of the feedlot, the odds of detecting *E. coli* were 9- to 13-times higher for distances ≤36 m (120 ft), but beyond 36 m (120 ft), the association was inconsistent. For example, the odds of detecting *E. coli* at distances of 37 to 128 m (121 to 420 ft) was not significantly different from >610 m (2000 ft) (Table 2). The months exhibiting a higher risk of *E. Coli* detection were generally January to March in all models. Lastly, when wind direction relative to the air sampler and feedlot was categorized as two 180° partitions of either upwind or downwind of the feedlot, the odds of detecting *E. coli* were 2.2- to 2.8-times greater for air samples taken downwind of the feedlot compared to air samples taken upwind of the feedlot. In contrast, when wind direction was categorized as 90° partitions of either (1) downwind, (2) side or upwind of the feedlot, or (3) little to no wind (<1.8 m/s or <4 mph), in this case, wind direction relative to the feedlot was not significant for Model A2 (in situ weather), but it was highly significant for Model A4 (weather station). Specifically, air samples located downwind of the feedlot and during little to no wind had substantially higher odds of *E. coli* detection (OR of 5.4 and 9.5, respectively) compared to air samples taken upwind for Model A4. This disagreement regarding the importance of wind direction relative to the location of a feedlot for detecting airborne *E. coli* is troubling given the motivation of this project was to better understand the risk of airborne bacterial contamination of in-field produce in proximity to a feedlot.

**Figure 5:**
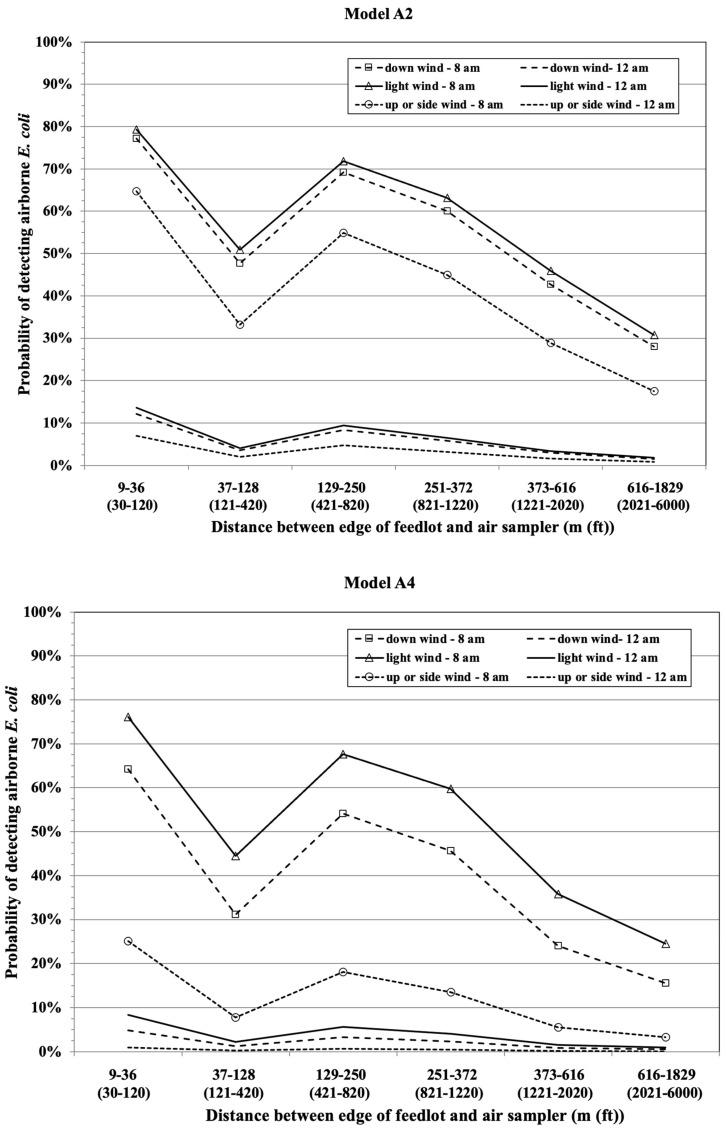
Predicted probability of detecting airborne *E. coli* as a function of distance to a commercial feedlot, Models A2 and A4 used in situ and a nearby weather station for meteorological data, respectively. Note: Models are with wind direction, presence of dust-generating activity, and hour of the day in March, and relative humidity is set at the arithmetic mean of 38% for the sampling period of March. With the wind direction set at 0°, downwind from a feedlot was a 90° partition or 135° to 225° when windspeed was ≥1.8 m/s (≥4 mph); up and side wind from a feedlot included 90° partitions for upwind (315° to 45°) and both lateral side winds (45° to 135° and 225° to 315°) when wind speed ≥1.8 m/s (≥4 mph); and light wind was wind speed less than 1.8 m/s (4 mph).

To aid in the interpretation of the regression models in Table 2, Figure 5 is a graphical representation of Model A2’s and Model A4’s predicted probabilities of detecting airborne *E. coli* as a function of distance to a commercial feedlot in Imperial Valley, wind direction, presence of dust-generating activity, and hour of the day in March, with relative humidity set at the arithmetic mean of 38% for the sampling period of March. This composite figure readily demonstrates that time of day when a sample was taken was the dominant predictor for the presence of airborne *E. coli*, with the distance from the feedlot being a significant but secondary influence relative to time of day for both models, and with the direction of wind relative to the feedlot only being significant for Model A4 (Figure 5). It is important to note that these are tentative model predictions given that the data were collected by using a longitudinal observational study design, whereby risk factors were measured at the same time the wind samples were collected, thus making causal inferences somewhat speculative.

## 4. Discussion

The primary goal of this longitudinal study was to determine the occurrence of airborne bacterial pathogens and bacterial indicators in proximity to commercial beef cattle feedlots in a produce-growing region. Achieving this goal would allow for a better characterization of potential foodborne pathogen risks resulting from growing produce in varying proximities to livestock production systems such as cattle feedlots. Prior research has indicated that the prevalence or concentration of airborne bacterial pathogens such as *E. coli* O157:H7 and *Salmonella* were typically zero for studies on airborne bacterial transmission adjacent to livestock operations [3,6]. A finding of non-detection for airborne bacterial pathogens may not be surprising given that the concentration of commensal bacteria such as enteric *E. coli* is often very low per 100 to 1000 L of air. In other words, if commensal or indicator bacteria are at 10^3^ to >10^6^ higher concentrations in mammalian fecal or environmental matrices compared to bacterial pathogens such as *Salmonella* or *E. coli* O157:H7, it is not surprising to observe a very low or zero prevalence for airborne pathogens if the concentration of commensal or indicator bacteria in those same samples is only 1 to 50 CFU per 1000 L of air. For example, Glaize et al. (2021) found that, although 14% of air samples collected 10 to 122 m downwind of a dairy farm tested positive for indicator *E. coli*, none of these air samples tested positive for *Salmonella* or STEC [6]. Similarly, Berry et al. (2015) measured airborne indicator *E. coli* in mean concentrations ranging from 1.4 to 68 CFU/1000 L, at distances between 0 and 180 m from a cattle feedlot, but none of these samples tested positive for *E. coli* O157:H7 [3].

This prior work suggests that sample air volumes should be maximized in order to detect what may be very low concentrations of airborne bacterial pathogens, if present; hence, we processed 6000 L of air per sampling event (rate of 100 L of air/min). Despite this larger volume of processed air, none of the 300 air samples collected over 6 months in proximity to five commercial feedlots in Imperial County, California, had either *E. coli* O157, non-O157 STEC, or *Salmonella* based on detection of qPCR-confirmed colony cultures from enriched samples. This suggests that the 300 air samples, which totaled 1.8 million liters of ambient air, were likely negative for these pathogens, resulting in an estimated maximum concentration of these bacterial pathogens being less than 1 CFU per million L of sampled air (1 CFU/1.8 × 10^6^ L = 0.56 × 10^−6^ CFU/L). Supporting this assertion of low airborne-pathogen levels near Imperial Valley feedlots was the observation that the mean concentration of *E. coli* for air samples taken at the same time and place in Imperial Valley was 0.17 CFU/6000 L of air, or ~28 CFU/10^6^ L of air (Table 1). As argued above, in prior work, we have observed >1000-fold higher concentrations of commensal or indicator bacteria per pathogen CFU in various environmental matrices. For example, in river-water samples from the Sacramento and San Joaquin Rivers, there were on average 6139 CFU of *E. coli* per one *Salmonella* CFU [15]; in six large reservoirs in Central California, we observed a mean concentration of 942 CFU fecal coliforms/100 mL, yet only 1.2% of these 257 water samples tested positive for *E. coli* O157:H7 [16]. It is likely that a much higher mean concentration of background commensal or indicator bacteria would need to be observed in these air samples in order to consistently detect a non-zero prevalence of airborne bacterial pathogens such as *E. coli* O157:H7 or *Salmonella*, especially if the predominant source of these airborne bacteria is due to fugitive dust from dry livestock manure on feedlot pen floors.

An additional conclusion from these negative data would be to calculate the likely maximum prevalence of airborne pathogens given the 300 pathogen-negative air samples. In other words, what is the probability that a small flux of airborne pathogens was randomly missed during air sampling? For this calculation, assume the 300 air samples comprise 1800 aliquots of 1000 L of air if we choose a 1000 L volume as an independent unit of air. Given that it takes about 10 min to process 1000 L of air using the MAS-100 Eco microbial air samplers (Merck KgaA, Darmstadt, Germany), we observed during this study that wind speed, wind direction, feedlot cattle behavior, and even dust-generating activities such as roadway vehicular traffic can all fluctuate over the course of 10 min while standing next to a commercial feedlot. This dynamic nature of ambient air suggests that the microbiology in each sequential 1000 L packet of air is quasi-independent from that of the others, especially when wind direction and wind speed dynamically change. Under these assumptions, the binomial distribution can be used to estimate the probability of observing zero positives in 1800 units of air given a maximum prevalence of the target pathogens per unit of air (1000 L/unit), and one will reject values for this maximal prevalence if the probability of observing 300 negatives is less than, for example, 10%. Therefore, if the underlying true prevalence for any one of these pathogens per 1000 L of air is either 0.001, 0.002, or 0.003, then there is an 17%, 3%, and <0.5% probability to observe zero positives among 300 samples, respectively. Based on this formulation, it is unlikely that the prevalence of *E. coli* O157, non-O157 STEC, or *Salmonella* in 1000 L air samples exceeds 0.002 (0.2%), or else we would have observed at least one positive sample for one or more target pathogens in this collection of *n* = 1800 units of 1000 L/unit of tested air. Interestingly, this estimated 0.2% maximum prevalence of airborne pathogens per 1000 L of air agrees with the estimated maximum concentration of pathogens per 10^6^ L of air (maximum 1 CFU/10^6^ L = 1 CFU/1000 units with 1000 L of air/unit = 0.1% prevalence per unit of air).

Although we did not detect *E. coli* O157, non-O157 STEC, or *Salmonella* based on qPCR-confirmed cultures from enriched samples, 5.3% and 0.3% of the initial enrichments from the 300 air samples were qPCR-positive for *stx* genes 1/2 and *Salmonella*, respectively. None of these suspect positives had a subsequent qPCR-confirmed colony on selective agar, and, therefore, they were classified as negative. Regarding the suspect *Salmonella* from a single air sample >1000 feet downwind from a feedlot, either this was a false qPCR-positive from the initial enrichment or the process of colony isolation on XLT4 agar resulted in loss of the *Salmonella* colony(ies) due to inhibition and/or low concentration in the enrichment broth. Regarding the sixteen samples that were qPCR-positive for *stx* genes 1/2, no STEC colonies were qPCR-confirmed from these suspect samples. This lack of STEC confirmation may have occurred because all nine were false qPCR-positives at enrichment, the concentration of STEC was very low in the enrichment broth, and therefore the colony missed on Chromagar STEC, or the *stx* gene was associated with an entity such as a bacteriophage or a bacterial specie that is not *E. coli*.

Speculating further on this suggestion of *stx* genes being present in non–*E. coli* background bacteria as the reason for observing qPCR-positives but negative for STEC, we did observe, on average, ~1900 cfu of total aerobic plate counts (APC) from the 6000 L air samples during the 6 months of this study (data not reported), indicating that there were numerous culturable aerobic bacteria being transported in the air during this study. It is well established that the majority of environmental microbes cannot be cultured [17] and that the proportion of cultivable microbes is only a small proportion of the total airborne microbial population [18]. For example, Li et al. (2020) found that culturable fractions of airborne bacteria ranged from less than 1.5% to 16.2% [19], with this fraction being relatively higher for air samples from livestock farms (10.9%) compared to air samples from other land uses, such as streets, gardens, lakes, and cropland. Hence, the total cfu of background airborne bacteria (culturable and non-culturable) in this region of Imperial Valley may have been 10-fold higher than the ~1900 APC/6000 L air we observed. It is not uncommon to observe *stx* genes 1 or 2 in environmental samples given that these genes have been associated with Gram-negative non–*E. coli* bacterial species, such as *Enterobacter cloacae* [20] and *Citrobacter rodentium* [21]. Lastly, the spatial pattern of *stx*-gene positives in these air samples was not associated with proximity to the feedlots, which is evident for the fixed sites at LGMA-guidance distances and also for sites with randomized distances (Table 1). This apparent ubiquitous occurrence of positives for airborne *stx* genes relative to feedlot proximity suggests that the source(s) of these airborne *stx* genes are more widely distributed in the surrounding farmland, including up to a mile distance from a feedlot. For example, the percentage of air samples testing qPCR-positive for stx genes 1/2 at ~122 m (~400 ft) and ~1609 m (~1 mile) from a feedlot were 8.3% and 10.0%, respectively.

Previous studies on airborne bacteria in proximity to livestock operations have generally used meteorological data from a nearby weather station [3,22,23,24]; in contrast, our study collected both in situ and nearby weather station meteorological data to compare the impact of data source on the final regression models for environmental factors associated with the presence of airborne *E. coli*. There were significant differences between these two data sources for ambient air temperature, wind speed, and wind direction during the days we sampled in Imperial Valley. Nevertheless, the pairs of regression models (pair Models A1 and A3; pair Models A2 and A4) that share the same coding for position of air sampler relative to wind and feedlot had similar sets of significant environmental variables associated with *E. coli* (Table 2). One notable exception was the lack of significance for the variable “position of air sampler relative to wind direction and feedlot” for Model A2. Given that sample hour and month, relative humidity, distance from feedlot, and the presence of dust generating activity were controlled for in the regression model, there was not a significant difference in the likelihood of detecting *E. coli* between air samples located during light wind conditions, downwind of the feedlot, and upwind or alongside the feedlot (Table 2). This variable regarding the position of the air sampler relative to wind and feedlot direction was significant for the other three models; hence, it may be prudent not to dismiss the significance of wind direction and feedlot position for evaluations focused on airborne indicator *E. coli*.

The significant variables in the four logistic regression models shown in Table 2 reveal numerous environmental associations with airborne indicator *E. coli* in proximity to commercial feedlots. For example, air samples in closer proximity to a feedlot compared to distances in excess of 610 m (2000 ft) were generally at higher odds for the presence of indicator *E. coli*, but this association was not consistent for some categories of distance (e.g., 37–128 m (121–420 ft) versus >610 m (>2000 ft) not different), and especially for the LGMA-guidance distances of 122 m (400 ft), 366 m (1200 ft), and 1609 m (1 mile) which did not demonstrate a trend for reduced airborne *E. coli* as a function of distance from feedlot (Table 1). Air samples taken during morning compared to afternoon (all four models), combined with low relative humidity (all four models), and in the presence of dust-generating activity (only two models) were associated with higher odds for airborne *E. coli*. Cattle can be more active in the early morning or late evening due to cooler temperatures compared to midday [4], and this may function to aerosolize dried manure and soil in the pens. Low relative humidity can be conducive to the formation of fugitive dust [25] containing indicator *E. coli* from the surface of dry agricultural fields and feedlot pens [26,27] that is then aerosolized during dust-generating activity such as vehicular traffic on dirt roads, cattle activity in the feedlot, or plowing fallow agricultural fields [28]. Other studies have found that increases in relative humidity and solar radiation were associated with reductions in the concentration of airborne bacteria [29,30]. In contrast, dehydrating environmental conditions, such as high air temperature and low relative humidity, can be unfavorable for microbial survival [31]. Lastly, a prior study concluded that there was no significant association between relative humidity or air temperature and airborne-microorganism concentrations from a dairy CAFO [23].

The association between low wind speed and airborne *E. coli* was significant for two of the four models (Table 2); specifically, the odds of detecting *E. coli* decreased 0.63-times for each additional meter/second of wind speed (Model A3) and increased 9.5-times during light wind conditions compared to being located either upwind or perpendicular to a feedlot when wind speed was ≥1.8 m/s (≥4 mph, Model A4). Although higher wind speeds can suspend small particles (with attached bacteria) in the air [27], aerosolize solid particulate matter [32], and also generate fugitive dust from dry livestock feces [33], it would appear that, in our study, the higher wind speeds resulted in atmospheric dilution and a subsequent decrease in the concentration of airborne bacteria [34]. This suggests that airborne *E. coli* is source-limited during conditions of higher wind speed for this region of California, whereby large volumes of air caused by wind quickly result in significant bacterial dilution, which apparently exceeds the rate of wind-driven fugitive dust generation and aerosolization of *E. coli* from sources of indicator *E. coli* in Imperial Valley.

Lastly, it is important to note that these results may not be generalizable outside of the Imperial Valley due to the unique environmental and management conditions common to this arid region.

## 5. Conclusions

These findings, when summarized together, indicate that windy days are not the high-risk periods for airborne *E. coli*; instead, it is early morning with low relative humidity and possibly low-to-no wind, combined with dust-generating activity (e.g., plowing a field, elevated vehicular traffic, and agitated cattle during feeding) that defines the environmental conditions of higher risk for airborne indicator *E. coli*. Interestingly, these are the environmental conditions that can occur when produce is harvested very early in the morning for this region of California, with the exception of the higher relative humidity in the early morning compared to the midday in the Imperial Valley. The elevated odds for airborne *E. coli* during these environmental conditions were further increased for unknown reasons during the months of January through March for this region of California (Table 2). Lastly, proximity to feedlots was also associated with an elevated odds of airborne *E. coli*, with substantially higher odds for air samples taken within 36 m (120 ft) of a feedlot compared to air samples taken >610 m (>2000 feet) away (odds ratios of 8.7 to 13.2; see Table 2); however, this association was surprisingly not evident for the critical LGMA-guidance distances stated for proximity to a compost facility (>122 m, >400 ft) or proximity to a CAFO with >1000 head (>366 m, >1200 ft). Despite these higher odds of detection for indicator *E. coli* in close proximity to a feedlot, the very low bacterial concentrations measured for ambient air (mean of 0.17 CFU of *E. coli*/6000 L) predicted the lack of detection of airborne bacterial pathogens in a total of 1.8 million L of air for this study, with an estimated mean maximum concentration for either *E. coli* O157, non-O157 STEC, or *Salmonella* being less than ~1 CFU per million L of air. These results may not be generalizable outside of the Imperial Valley due to the unique environmental and management conditions common to this arid region. It is unclear at this time what level of additional food safety risk is incurred by growing produce exposed to very low levels of airborne bacteria, but additional studies are underway which aim to clarify the levels of food safety risk being generated when leafy green produce is grown in Imperial Valley under these environmental conditions [35].

## Figures and Tables

**Table 2:** Logistic regression model for the association between indicator of airborne *E. coli* and environmental variables in proximity to 5 commercial feedlots in Imperial Valley, California (November 2020–April 2021).

Model Variable	Level of Categorical Variable	In Situ Kestrel				Weather Station			
		Coef.	*p*-Value	OR	95% CI	Coef.	*p*-Value	OR	95% CI
		Model A1				Model A3			
Hour sample was taken		−0.87	<0.01 *	0.42	(0.30, 0.58)	−0.88	<0.01 *	0.41	(0.29, 0.59)
(1–24 h)									
Rel. humidity (0–100)		−0.04	0.02 *	0.96	(0.93, 0.99)	−0.03	0.02 *	0.97	(0.94, 0.995)
Wind speed (m/s)		NS				−0.47	0.01 *	0.63	(0.44, 0.90)
Dust-generating activity ^a^	Present	0.94	0.049 *	2.57	(1.01, 6.56)	NS			
	Absent ^b^	0	-	1	-				
Distance between feedlot edge and air sampler m (ft)	9–36 (30–120)	2.22	0.01 *	9.21	(1.65, 51.38)	2.58	<0.01 *	13.23	(2.25, 77.87)
	37–128 (121–420)	0.94	0.25	2.55	(0.52, 12.46)	1.19	0.15	3.3	(0.66, 16.48)
	129–250 (421–820)	1.77	0.04 *	5.88	(1.11, 31.04)	2.09	0.02 *	8.08	(1.45, 45.10)
	251–372 (821–1220)	1.46	0.06	4.29	(0.97, 18.99)	1.63	0.04 *	5.12	(1.12, 23.41)
	373–616 (1221–2020)	0.59	0.58	1.81	(0.22, 14.80)	0.63	0.56	1.88	(0.22, 16.00)
	616–1829 (2021–6000) ^b^	0	-	1	-	0	-	1	-
Month when sampling occurred	November 2020	1.38	0.1	3.96	(0.78, 20.14)	0.17	0.84	1.18	(0.23, 6.22)
	December 2020	0.83	0.27	2.3	(0.52, 10.13)	0.31	0.7	1.36	(0.29, 6.52)
	January 2021	2.09	<0.01 *	8.06	(2.02, 32.14)	1.43	0.05 *	4.17	(1.03, 16.83)
	February 2021	1.71	0.01 *	5.53	(1.46, 20.92)	1.12	0.1	3.07	(0.80, 11.81)
	March 2021	2.22	<0.01 *	9.23	(2.43, 35.07)	1.69	0.01 *	5.44	(1.45, 20.47)
	April 2021 ^b^	0	-	1	-	0	-	1	-
Position of air sampler relative to feedlot and wind direction (180° partitions)	Downwind	0.79	0.045 *	2.2	(1.02, 4.76)	1.03	0.01 *	2.79	(1.34, 5.82)
	Upwind ^b^	0	-	1	-	0	-	1	-
		Model A2				Model A4			
Hour sample was taken		−0.8	<0.01 *	0.45	(0.33, 0.62)	−0.89	<0.01 *	0.41	(0.29, 0.59)
(1–24 h)									
Rel. humidity (0–100)		−0.04	0.03 *	0.96	(0.93, 0.997)	−0.04	0.01 *	0.96	(0.93, 0.99)
Distance between feedlot edge and air sampler m (ft)	9–36 (30–120)	2.16	0.01 *	8.7	(1.58, 48.02)	2.28	0.01 *	9.74	(1.70, 55.83)
	37–128 (121–420)	0.85	0.28	2.35	(0.49, 11.21)	0.9	0.27	2.46	(0.50, 12.02)
	129–250 (421–820)	1.75	0.04 *	5.76	(1.11, 29.83)	1.86	0.03 *	6.43	(1.20, 34.42)
	251–372 (821–1220)	1.35	0.07	3.87	(0.89, 16.75)	1.52	0.05	4.59	(1.03, 20.52)
	373–616 (1221–2020)	0.65	0.54	1.91	(0.24, 15.29)	0.54	0.61	1.71	(0.21, 13.85)
	616–1829 (2021–6000) ^b^	0	-	1	-	0	-	1	-
Month when sampling occurred	November 2020	0.95	0.26	2.59	(0.50, 13.48)	0.11	0.9	1.12	(0.21, 6.04)
	December2020	0.6	0.44	1.83	(0.39, 8.57)	0.5	0.54	1.66	(0.33, 8.29)
	January 2021	1.86	0.01 *	6.4	(1.55, 26.41)	1.72	0.02 *	5.59	(1.32, 23.69)
	February 2021	1.49	0.03 *	4.46	(1.18, 16.83)	1.44	0.04 *	4.21	(1.04, 17.00)
	March 2021	2.07	<0.01 *	7.92	(2.10, 29.90)	2.1	<0.01 *	8.14	(2.10, 31.62)
	April 2021 ^b^	0	-	1	-	0	-	1	-
Position of air sampler relative to feedlot and wind direction (90° partitions)	Downwind	0.61	0.17	1.84	(0.77, 4.44)	1.68	0.01 *	5.38	(1.45, 19.94)
	Light wind ^c^	0.74	0.13	2.10	(0.80, 5.52)	2.25	<0.01 *	9.46	(2.83, 31.68)
	Up or side wind ^b,c^	0	-	1	-	0	-	1	-
Dust-generating activity ^a^	Present	0.98	0.04 *	2.67	(1.03, 6.92)	NS			
	Absent ^b^	0	-	1	-				

Coef. = beta coefficient from logistic regression model, OR = odds ratio, CI = confidence interval, Rel. humidity = relative humidity. * Represents *p* ≤ 0.05. ^a^ Dust-generating activity is coded present when one or more of the following events occur within 400 m (¼ mile) during the air-sampling time period: vehicular traffic, plowing field, feedlot cattle exhibiting high levels of movement, cattle or sheep grazing, farming activity in the adjacent land of sampling sites, and/or biomass burning. ^b^ Referent category for calculating the odds ratio (OR). ^c^ Light wind is wind speed less than 1.8 m/s (4 mph); up or side wind includes 90° partitions for upwind and both lateral side winds with wind speed ≥ 1.8 m/s (≥4 mph).

## Data Availability

The raw data show all air samples, along with microbiological results and environmental measurements. These samples were used for statistical analysis of the association between indicator *E. coli* and risk factors.
